# Measures of cardiovascular autonomic activity in insomnia disorder: A systematic review

**DOI:** 10.1371/journal.pone.0186716

**Published:** 2017-10-23

**Authors:** Marina-Marinela Nano, Pedro Fonseca, Rik Vullings, Ronald M. Aarts

**Affiliations:** 1 Department of Electrical Engineering, Eindhoven University of Technology, Eindhoven, The Netherlands; 2 Sleep Medicine Centre Kempenhaeghe, Heeze, The Netherlands; 3 Philips Research, High Tech Campus, Eindhoven, The Netherlands; TNO, NETHERLANDS

## Abstract

**Background:**

Insomnia disorder is a widespread sleep disorder with a prevalence of approximately 10%. Even though the link between insomnia and cardiovascular activity is not exactly clear, it is generally assumed that cardiovascular autonomic modifications could occur as a result of sleeplessness, or, alternatively, that autonomic alterations could be an expression of a hyper-arousal state. This review investigates whether cardiovascular measures are different between insomniacs and controls.

**Methods:**

Electronic databases were systematically searched, and 34 studies were identified. Heart rate variability features, the association of cardiac and EEG activity, physiologic complexity measures, and cardiovascular activity, assessed by measures such as pre-ejection time, blood pressure, and heart rate dynamics were studied. Given the heterogeneity of the studies, a narrative synthesis of the findings was performed.

**Results:**

This review study found overall differences in cardiovascular activity between insomniacs and controls in most of the observational studies (21/26), while the expression of cardiovascular regulation varied between the examined insomniac groups. All the studies that investigated the association of cardiac activity and EEG power reported an altered relation between autonomic activity and EEG parameters in insomniacs.

**Conclusion:**

Autonomic regulation tends to be consistent between insomniacs, as long as they are grouped according to their respective phenotype, as shown in the insomnia subgroup with objectively short sleep duration. Our hypothesis is that these differences in the expression of cardiovascular activity could be explained by the heterogeneity of the disorder. Therefore, the determination of insomnia phenotypes, and the study of cardiovascular measures, rather than heart rate variability alone, will give more insight into the link between insomnia and cardiovascular regulation. This study suggests that cardiovascular activity differs between insomniacs and controls. These new findings are of interest to clinicians and researchers for a more accurate insomnia assessment, and the development of personalized technological solutions in insomnia.

## Introduction

Difficulties initiating or maintaining sleep are very prevalent sleep complaints in the general population [1, 2]. If sleeplessness meet specific diagnostic criteria the term insomnia disorder is used. Multinational studies that used the Diagnostic and Statistical Manual of Mental Disorders fourth edition (DSM-IV) criteria reported prevalence rates of insomnia disorder that range from 3.9% to 22.1%, with an average of approximately 10% [3]. This is a broad range that reflects different modalities of investigation and the population under study [1]. Information from new studies on the prevalence of insomnia disorder using DSM-V criteria, is currently limited.

On the latest update of DSM, insomnia disorder is defined as a predominant complaint of dissatisfaction with sleep quality or duration and is accompanied by difficulties in initiating sleep at bedtime, frequent or prolonged awakenings, or early-morning awakening, with an inability to return to sleep [4]. This sleep disturbance causes clinically significant social, occupational, educational, academic, and behavioral distress or impairment. These difficulties occur despite adequate opportunity for sleep. Diagnosis of insomnia is made when sleep difficulties are present for 3 or more nights per week, and last for more than 3 months [4]. Thus, insomnia is a condition characterized by both nocturnal and diurnal symptoms.

The cardiovascular autonomic nervous system (ANS) appears to be closely linked to sleep and circadian physiology, as demonstrated by the disrupted autonomic control that accompanies sleep loss [5]. Additionally, autonomic activity is integrated with cognition and emotion, among others [6]. Sleep loss or deficiency can usually occurs as a result of sleep deprivation, sleep fragmentation, or difficulty of falling asleep. In insomnia disorder sleep loss is usually caused by difficulties of maintaining (fragmented sleep) or initiating sleep. In addition to sleep loss, insomnia disorder is frequently accompanied by various changes, such as cognitive arousal/stress, degraded mood, depression or anxiety and fatigue [7]. To date, two major hypotheses have been made about the link between autonomic function and insomnia [8]. According to the first hypothesis, autonomic modifications could occur as a result of sleep fragmentation [9]. This hypothesis is supported by studies showing that autonomic arousals without cortical involvement are an epiphenomenon of sleep fragmentation and altered sleep continuity [9]. Furthermore, autonomic sleep fragmentation has been linked to diurnal increase in sympathetic activity and elevated blood pressure (BP) in healthy elderly [10]. According to the second hypothesis, autonomic alterations could be an expression of a hyper-arousal state [8]. Evidence of an increase in heart rate (HR) and the absence of a normal drop in autonomic activity during falling asleep, along with alterations of other physiologic parameters (e.g. body temperatures, stress hormones), could be considered indicators of a state of arousal that predisposes the individual to poor sleep [11–13]. Therefore, relevant physiology data obtained by cardiovascular ANS measures may provide new insight into the link between insomnia disorder and cardiovascular autonomic activity.

While two reviews [14, 15] examining heart rate variability (HRV) and one review [16] investigating cardiovascular dysfunction between normal sleep and sleep disorders have been published, their primary focus was not insomnia disorder, so findings regarding insomnia were not methodically incorporated. One review [17] focusing exclusively on HRV and insomnia was recently published. Cardiovascular activity, compared to HRV alone, provides a more complete overview of the autonomic activity. For instance, studies have shown that the use of HRV for the estimation of autonomic regulation has limitations [18] and additional diagnostic value can be obtained from measures such as pre-ejection period (PEP) [19]. In this study, we do not restrict the review to HRV, as Dodds et al. [17] did, but also incorporate other cardiovascular measures of autonomic activity, such as PEP, cardiopulmonary coupling (CPC), left ventricular ejection time, BP and HR slope for the analysis of HR dynamics in order to investigate whether cardiovascular activity measures are different between insomniacs and controls. In addition, we aim to examine how interventions influence cardiovascular activity.

## Methods

### Search strategy

For this review, the PubMed and Scopus electronic databases were systematically searched for articles published until 9th of October 2016, using keywords to identify all studies specifically designed to define cardiovascular differences between insomniacs and healthy controls. A search of publications was conducted using the following medical subject headings or key words: “heart rate”, “cardiac”, “cardio”, “blood pressure”, “autonomic”, “sympathetic”, “parasympathetic”, “arterial”, “vascular”, “baroreflex” and “insomnia”. Based on search options provided by the two electronic databases, the search approach was adjusted as shown in S1 Appendix. To ensure literature saturation, we examined the reference lists of the included papers and of the relevant reviews which were identified by the search. The literature search was limited to studies conducted with human participants, published in the English language.

### Study selection

In order to identify relevant publications, the following criteria were applied in the initial stages of the scrutiny process: (1) participants were adult (≥ 18 years old), (2) studies include adults participants diagnosed with insomnia (observational) or treated for insomnia (interventional), (3) comparison of insomniacs with control group (observational studies) or same group of insomniacs before and after intervention (interventional studies), and (4) observational studies include non-invasive techniques, but not in vitro tests, such as saliva test. Articles meeting these criteria were collected and data was extracted for analysis by the first author. After duplicate removal studies were reviewed for eligibility using title, abstract and full text when it was required.

### Cardiovascular measures used to explore autonomic changes and their physiological significance interpretation

In this section, cardiovascular measures used in literature to investigate and study autonomic changes are introduced. Additionally, their physiological interpretation is presented.

Over the past years, different methods of cardiovascular autonomic activity and HRV quantification have been developed, such as frequency, time-frequency, temporal, geometrical, and nonlinear analysis [20]. Autonomic cardiovascular measures can be examined traditionally through the quantification of average HR and BP [5] and more recently through non-linear approaches by using detrended fluctuation analysis (DFA), [21] entropy derived [15, 22–24], Poincaré Plot [25], and Lempel-Ziv [26, 27] measures. As described in detail previously [5], HR and BP variations can be expressed by the standard deviation around the mean, or by their rhythmic and non-rhythmic components. RR time series (the time elapsed between two successive R-waves of the QRS complex on the electrocardiogram (ECG)) and BP also show short-term oscillations in a frequency range between 0 and 0.5 Hz. Traditionally, HRV and cardiovascular parameters are measured in the time and frequency domain.

Standard HRV analysis has been well summarized by the task force of the European society of cardiology [28]. The most commonly used time domain measures are described in Table 1. Rate pressure product (RPP) is an index of the overall cardiac workload [29], and is calculated as follows: HR * systolic BP/100 [30]. HR and BP physiologically decrease at night, compared to during the day. Systolic BP reduction at least 10% during sleep, compared to daytime, is commonly referred to as “dipping”. The PEP is influenced by sympathetic activity [5].

**Table 1:** Summary of time domain cardiovascular measures and their physiological interpretation.

Feature	Description	ANS interpretation	Study
PEP	the time from the onset of the ECG Q-wave to the opening of the aortic valve	a marker of beta-adrenergic sympathetic activity	[12, 59–64]
RPP	the product of HR and SBP	index of the overall cardiac workload	[63]
SDNN	standard deviation of RR or NN intervals for a desired period and is measured in ms	both sympathetic and parasympathetic activity and therefore provides an index of overall HRV	[8, 13, 47, 60, 64–67]
RMSSD	square root of the mean squared differences of successive NN intervals for a desired period, measured in ms	parasympathetic activity	[8, 13, 47, 60, 61, 64–67]
pNN50	percentage of successive NN intervals that differ more than 50 ms	parasympathetic activity	[8, 13, 60, 64–66]

Abbreviations— ANS: Autonomic nervous system, ECG: electrocardiogram, HRV: heart rate variability, ms: milliseconds, PEP: pre-ejection time, RPP: rate pressure product, RR time series: the time elapsed between two successive R-waves of the QRS complex on the electrocardiogram, study: represents the studies that the feature was used, *Note:* *Abbreviation not mentioned here are described in the “Description” column of the table.*

In the frequency domain, HRV is evaluated by spectral analysis. As described in detail previously [5, 28, 31], spectral analysis of RR intervals and BP variability gives information on how power of the signal is distributed as a function of the frequency. Kay and Marple presented an extensive summary of several techniques used for spectral analysis [32]. Methods for power spectral density estimation can be generally classified as non-parametric and parametric [28]. The two most common approaches [33, 34] used for spectral analysis of RR time series are Fourier transform (FFT) [31] and autoregressive model (AR) [35]. The high frequency power (HF) components (0.15-0.4 Hz) reflect the respiration-driven modulation of sinus rhythm, and have been used as an index of tonic vagal drive [5, 28, 31, 34, 36, 37]. The physiological significance of the very low frequency (VLF) component is still unclear, and limited data suggest that it might reflect vagal and rein-angiotensin system effects on HR [14, 28, 31, 34]. The physiological interpretation of the low frequency (LF) power components (0.04-0.15 Hz) is controversial. Some studies [28, 31, 38] support the conclusion that LF power is considered to reflect both sympathetic and vagal modulation of the heart, while other studies [5, 39] indicate that it might be an index of the baroreflex sensitivity (BRS) for control of HR. Moreover, for some researchers [28, 37, 40, 41], LF is seen as a marker of sympathetic modulation, particularly when it is expressed in normalized units. LF rhythm can also be modulated by irregular breathing patterns [5]. Consequently, the LF/HF ratio is considered by some researchers to express sympatho-vagal balance, and by others, to reflect only sympathetic modulations [28]. It should be noted that Eckberg et al. [42] questioned the use of the LF as an indicator of sympatho-vagal tone balance. As described previously [28], these disagreements in the interpretation of LF can be attributed to the fact that several conditions associated with sympathetic activation, can cause a decrease in the absolute power of the LF component. For example, during sympathetic activation, tachycardia follows, and is usually characterized by a reduction in total power, while the opposite happens during vagal activation [28]. In this way, when the LF is measured in milliseconds squared, the variations in total power affect LF and HF in the same direction(for details see [28]). Due to the reduction in total power, LF could remain unaltered if it is measured in milliseconds squared. Nevertheless, if normalization is performed, an increase in LF becomes more evident [28]. Other reasons that could explain this discrepancy include the fact that respiration parameters and behavior are influenced by age and activity, among others. For instance, during tasks, individual differences might exhibit a wide range of spontaneous breathing rates, which may result in a contribution to the HF band by individuals with faster breathing frequencies, and a contribution to the LF band by individuals with slower breathing frequencies [43].

Regarding BP variability, LF_*BP*_ components in systolic BP variability are considered an index of efferent sympathetic vascular modulation, whereas the HF_*BP*_ components express mechanical effects of respiration on blood pressure changes [5]. BRS regulates BP in order to preserve stability. BRS can be measured by either provocation of the carotid baroreceptors with phenylephrine or by the spectral technique which quantifies spontaneous fluctuations of the systolic blood pressure spectral power and the corresponding RR time series spectral power in different frequency bands [44–46]. The latter approach was first introduced by Robbe et al. [45] and is used by the authors of the reviewed studies [47]. The *α*BRS is computed using the square root of the ratio of RR time series and systolic BP power spectra in the LF and HF bands (*α*LF and *α*HF) [47–49]. The *α*-index is computed only when the squared coherence function (*k*^2^) of the systolic BP and RR time series exceeded 0.56 [47–49]. *α*Total is defined as the mean of *α*LF and *α*HF. TF-BRS (the evaluation of the transfer function between time series of systolic BP and RR time series) is computed by averaging the gain function in the LF band regardless of a given coherence between systolic BP and RR time series [47, 48, 50]. The *α*LF describes the gain of the relation between the BP and RR time series power spectra in the LF band [47, 48, 50]. The *α*LF component describes the gain in the spectral band of the respiration frequency. *α*Total gives an assessment of the overall baroreceptor gain [51].

Recently, new methods have been used for the analysis of HRV, in order to consider the non-stationary characteristics of the ECG signal and the non-linear fluctuations in HR. These techniques attempt to characterize cardiovascular ANS in terms of regularity and complexity, based on information carried by RR time series through the use of entropy derived and Lempel-Ziv measures [22, 23, 26, 27]. Sample entropy is the negative logarithm of conditional probability of the sequences of RR time series. High sample entropy shows that there is a low probability of repeated sequences in the RR time series, which means lower regularity and more complexity in the RR time series [22, 52]. Multiscale entropy is estimated based on the computation of the sample entropy over a range of temporal scales [23] (For more details about sample and multiscale entropy see [22, 23]). The Lempel-Ziv complexity algorithm provides information regarding the complexity of RR time series [27]. Complexity is related to the number of distinct patterns along the RR time series and the rate of their occurrence within a given sequence [26]. (For more details regarding the Lempel-Ziv complexity algorithm and the coding procedure used in the reviewed studies see [53, 54]). Detrended fluctuation analysis (DFA) examines the fractal scaling properties of HR fluctuations in the non-stationary RR time series on different time scales for the detection of long-range correlation between the RR intervals [21]. CPC was introduced by Thomas et al. [55] as the product of the coherence and cross-spectral power of the RR or NN time series and the ECG-derived respiratory time series. ECG-spectrographic variables were found to correlate strongly with EEG measures of sleep stability, suggesting that the resulting sleep spectrogram can classify sleep as “stable” (high-frequency coupling band (0.1—0.4 Hz) (HFC)) and “unstable” (low-frequency coupling band (0.1—0.4 Hz) (LFC)) [55–57]. The cardiovascular measures and their interpretation that are used in the reviewed studies are presented in Tables 1, 2 and 3. For details about the description and ANS interpretation columns of the Tables 1, 2, and 3 please see [5, 14, 21–23, 28, 47–51, 53–55, 58].

**Table 2:** Summary of nonlinear cardiovascular measures.

Feature	Description	Study
entropy of RR time series	a non-linear measure which examines the regularity of RR time series and it increases with greater degree of irregularity reaching a maximum at completely random system	[53, 68]
Lempel-Ziv complexity of RR time series	a non-linear measure that estimates the complexity of RR time series and quantifies the rate of new patterns along the sequence	[53]
DFA of RR time series	a non-linear measure that characterizes the pattern of variation and long-range correlations of RR time series across multiple time scales	[53]

Abbreviations— DFA: detrended fluctuation analysis, RR time series: the time elapsed between two successive R-waves of the QRS complex on the electrocardiogram, study: represents the studies that the feature was used, *Note:* *Abbreviation not mentioned here are described in the “Description” column of the table.*

**Table 3:** Summary of the frequency cardiovascular measures and their physiological interpretation.

Feature	Description	ANS interpretation	Study
Total power	variance of all RR or NN intervals measured in ms^2^		[59, 60]
VLF	Low frequency power (0.003—0.04 Hz) measured in ms^2^	parasympathetic activity and renin-angiotensin system effects on HR	[68]
LF	very low frequency power (0.04—0.15 Hz) measured in ms^2^	measure that includes both sympathetic and vagal influence*	[8, 11–13, 47, 59, 60, 64–67, 69–78]
LF_*norm*_	low frequency power (0.04—0.15 Hz) normalized using total power	marker of sympathetic modulation*	[8, 11–13, 47, 59, 60, 64–67, 69–78]
HF	high frequency power (0.15—0.4 Hz) measured in ms^2^	marker of parasympathetic/vagal activity	[8, 13, 47, 60, 64–67]
HF_*norm*_	high frequency power (0.15—0.4 Hz) normalized using total power	marker of parasympathetic/vagal activity	[8, 13, 47, 60, 64–67]
LF/HF	ratio of LF to HF	reflects sympatho/vagal balance or sympathetic modulations*	[8, 11, 13, 59, 60, 64–67, 69–71, 73–77]
1/f	slope of the power-low regression line of RR time series fitted to the power spectrum for f < 0.01 Hz		[53]
CPC	the product of the coherence and cross-spectral power of the RR or NN time series and the ECG-derived respiratory time series		[57]
*α*LF	the squared root of the ratio of RR time series and systolic BP power spectra in the (0.04-0.15 Hz) frequency band measured in ms/mmHg		[47]
*α*HF	the squared root of the ratio of RR time series and systolic BP power spectra in the (0.15-0.4 Hz) frequency band measured in ms/mmHg		[47]
*α*Total	mean of *α*LF and *α*HF measured in ms/mmHg		[47]

Abbreviations— ANS: autonomic nervous system, CPC: cardiopulmonary coupling, HRV: heart rate variability, mmHg: millimeter of mercury, ms: milliseconds, Total power: variance of all NN or RR intervals, study: represents the studies that the feature was used

*: the interpretation is controversial, *Note:* *Abbreviation not mentioned here are described in the “Description” column of the table.*

## Results

### Reviewed studies

**Fig 1:**
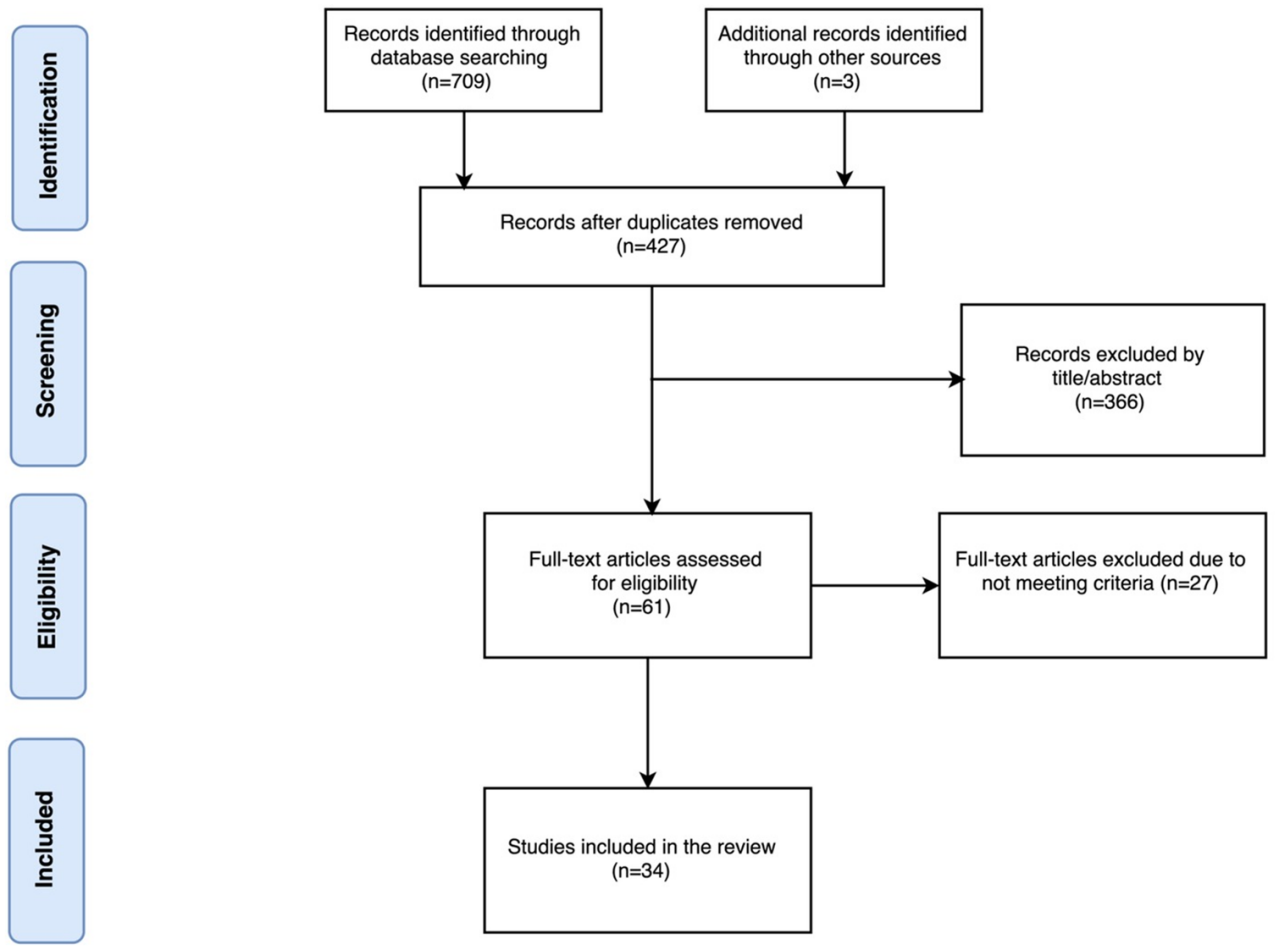
Study search and selection for measures of cardiovascular activity in insomniacs and controls. Modified PRISMA 2009 flow diagram [89].

The initial combined database search generated 709 records. After duplicate removal and English language restriction, 427 electronic records were identified and screened for eligibility. The manual search of reference lists of relevant papers and reviews identified three papers [53, 64, 72]. Three additional studies [79–81] were added to explain the transition from “poor” sleepers to clinically defined insomniacs. The vast majority of these articles (n = 366) were excluded by title or abstract alone (see Fig 1) based on criteria mentioned earlier. Full text articles were obtained for the 61 remaining articles. Ultimately, 34 studies were identified that met the inclusion criteria for this review. Twenty six observational studies ([8, 11–13, 47, 53, 57, 59–63, 65, 66, 68–73, 82–87]) and eight interventional studies ([64, 67, 74–78, 88]). Study details such as diagnostic criteria, demographics, number of participants, insomnia severity, and type of intervention are presented in Tables 4 and 5.

**Table 4:** Characteristics of the 26 observational studies.

Authors	Sample, Sex Age (mean± std, range)	Diagnostic criteria	Insomnia Severity	Sleep Efficiency (Insomniacs-Controls)	Monitoring and analysis details
Stepanski et al. [82]	Insomniacs: 24 M 34.5 ± 8.3, ND Controls: 25 M 34.0 ± 7.6, ND	DIMS (psychophysiological or idiopathic insomnia) and SE < 85% on one PSG night	ND	< 85%—> 90% (PSG of 1 night)	Monitoring time: during sleep and a morning task, ECG sampling rate: ND, Period of analysis: averaged over an hour, Spectral analysis technique: Not applicable
Bonnet et al. [11]	Insomniacs: 12 (M&F) 31.2 ± 6.8, ND Controls: 25 (M&F) 29.1 ± 5.2, ND	Subjectively reported insomnia for ≥ 4 nights per week for a year AND (SOL>30 min or SE<85%) on both PSG nights	Insomniacs subjectively reported: i) a sleep problem; ii) (SOL ≥ 45 min OR WASO ≥ 60 min) for ≥ 4 nights per week and iii) condition > 1 year	82±11%—93±3.2%	Monitoring time: during sleep, ECG sampling rate: 500 Hz, Period of analysis: 5 min, Spectral analysis technique: BMDP Tl Program
de Zambotti et al. [59]	Insomniacs: 9 (4 M) 23.0 ± 2.4, 20-26 Controls: 9 (4 M) 23.6 ± 3.2, 19-28	DSM-IV criteria for primary insomnia and subjectively reported insomnia history for ≥ 1 year	PSQI: 9.67 ± 1.67, AIS: 10.00 ±3.16, HS: 44.56± 4.03	91(±5)%—96(±3)%	Monitoring time: during sleep, ECG sampling rate: 500 Hz, Period of analysis: 2 min, Spectral analysis technique: FFT
de Zambotti et al. [60]	Insomniacs: 13 (8 F) 23.0 ± 2.4, 20-28 Controls: 14 (7 F) 23.6 ± 3.2, 23-28	Research Diagnostic Criteria for primary insomnia and (PSQI ≥ 5, ISI ≥ 11)	PSQI: 10.0 ± 2.0, AIS: 15.8 ±3.3, Length of insomnia (months): 58.5± 39.9	87.1(±8.4)%—94.0(±3.9)%	Monitoring time: during sleep, ECG sampling rate: 512 Hz, Period of analysis: 2 min in frequency domain and 5 min in time domain, Spectral analysis technique: FFT
Spiegelhalder et al. [13]	Insomniacs: 58 (36 F) 39.5 ± 11.8, ND Controls: 48 (27 F) 37.3 ± 11.4, ND	DSM-IV. For second analysis: INSD SE ≥ 85% vs ISSD SE < 85% based on PSG	PSQI: 11.2 ± 2.8, BDI: 9.7 ±6.6, Length of insomnia (years): 10.9± 9.9	85.8(±9.8)%—89.0 (±9.2)% (2nd PSG night)	Monitoring time: during sleep, ECG sampling rate: 400 Hz, Period of analysis: 5 min, Spectral analysis technique: FFT
Bianchi et al. [53]	Insomniacs: 11 (M&F) 39.5 ± 11.8, 35-50 Controls: 17 (M&F) 37.3 ± 11.4, 40-50	ND—subjects underwent polysomnographic evaluation	ND	ND	Monitoring time: during sleep, ECG sampling rate: 128 Hz, Period of analysis: ND, Spectral analysis technique: ND
Jurysta al. [69]	Insomniacs: 14 M 42 ± 12, 16-63 Controls: 14 M 41 ± 10, 16-55	DSM IV and ICSD-R (revised edition) for chronic primary insomnia, subjective complaints for ≥ 1 month, SE < 85%	ND clearly. Available sleep characteristics for revision	74(±9)%—90(±3)%	Monitoring time: during sleep, ECG sampling rate: 200 Hz, Period of analysis: 2 min, Spectral analysis technique: FFT
Farina al. [8]	Insomniacs: 85 (38 M) 53.2 ± 13.6, 27-81 Controls: 55 (23 M) 54.2 ± 13.9, 27-76	ICSD-2 for primary insomnia	Mean duration of insomnia > 2 years	82.4(±22)%—86.9(±14.2)%	Monitoring time: during sleep, ECG sampling rate: 256 Hz, Period of analysis: 5 min, Spectral analysis technique: Autoregressive Model
Mazza et al. [70]	Insomniacs: 6 (2 F) 53.3 ± 14.9, 34-70 Controls: 55 (32 F) 54.2 ± 13.0, 27-76	Diagnosis of chronic benzodiazepine abuse according to the DSM-IV-TR. In all cases patients were initially prescribed for the treatment of chronic insomnia	Validated Italian version of the PSQI>5, ESS>6	86.2(±8.5)%—92.0(±5.3)%	Monitoring time: during sleep, ECG sampling rate: ND, Period of analysis: ND, Spectral analysis technique: Autoregressive Model
Lanfranchi et al. [83]	Insomniacs: 13 (9 F) 42 ± 9, 30-60 Controls: 13 (9 F) 42 ± 7, 30-60	DSM-IV-R for chronic insomnia and meet the following criteria: Subjectively reported SOL and/or WASO > 30 min, TST < 6.5 h, SE < 85% ii) presence of insomnia ≥ 3 nights per week for ≥ 6 months iii) ISI ≥ 15	All subjects suffered from mixed (difficulties initiating and maintaining sleep) insomnia, ISI: 18.2 ± 2.1, BDI: 9.5 ± 2.1	65(±12)%—92(±7)%	Monitoring time: during daytime and sleep, ECG sampling rate: 256 Hz, Period of analysis: averaged over one hour, Spectral analysis technique: NA
Fang et al. [65]	Insomniacs: 18 (6 M) 34.2 ± 14.5, 20-60 Controls: 21 (7 M) 27.8 ± 8.7, 20-50	DSM-IV for primary insomnia and at least one of the following criteria on both actigraphy and diary measures: i) WASO >30 min, ii) TST ≤6.5 h, ii) SE ≤85%	Subjectively reported SOL and/or WASO > 30 min, TST < 6.5, SE < 85% b) presence of insomnia ≥ 3 nights per week for ≥ 6 months (c) ISI≥ 15.23	90.2(±4.8)%—96.9(±2.1)% (based on actigraphy)	Monitoring time: during daytime, ECG sampling rate: 500 Hz, Period of analysis: 5 min, Spectral analysis technique: FFT
Varkevisser et al. [61]	Insomniacs: 11 (6 M) 43.8 ± 8.9, 31-54 Controls: 13 (7 M) 44.9 ± 7.7, 33-53	ICSD-R for chronic psychophysiologic insomnia (with ambulatory PSG)	Available characteristics (based on actigraphy) for revision	73.3 (±3.6)%—82.3 (±6.7)% (based on actigraphy)	Monitoring time: during daytime, ECG sampling rate: 1000 Hz, Period of analysis: averaged over 30 sec segments, Spectral analysis technique: NA
Yang et al. [68]	Insomniacs: 47 (4 M) 41.6 ± 11.7, 23-63 Controls: 88 (4 M) 24.8 ± 2.7, 22-64	DSM-IV for primary insomnia	PSQI: 10.7 ± 3.9, BDI: 10.9 ± 6.5. Available characteristics for revision	ND	Monitoring time: during daytime and sleep, ECG sampling rate: ND, Period of analysis: ND, Spectral analysis technique: ND
Floam et al. [84]	Insomniacs: 29 (19 F) 25.3 ± 1.6, 18-55 Controls: 19 (13 F) 25.4 ± 1.4, ND	DSM-V for primary insomnia disorder	PSQI: 11.2 ± 0.7, insomnia duration (years): 6.4 ± 0.8, actigraphy based SOL: 27 ± 4, WASO: 30 ± 3, SFI: 15.5 ±1.4	ND	Monitoring time: during daytime, ECG sampling rate: NA, Period of analysis: average of the 5 measurements over a 15-min period Spectral analysis technique: NA
Covassin et al. [62]	Insomniacs: 8 (4 M) 22.9 ± 2.4, ND Controls: 8 (4 M) 24.8 ± 2.7, ND	DSM-IV for primary insomnia for at least 1 year and PSQI: ≥ 6, AIS ≥ 6, ISI: ≥ 11	Insomnia for at least 1 year and PSQI: 9.6 ± 1.3, AIS 10.8 ± 2.4, ISI: 14.0 ± 2.7, SOL: 16.9 ± 14.7, WASO: 27.1 ± 19.1	90.6(±5.1)%—96.3(±2.4)%	Monitoring time: during daytime, ECG sampling rate: 500 Hz, Period of analysis: averaged over 30 sec segments, Spectral analysis technique: NA
Jiang et al. [66]	Insomniacs: 55 (25 M) 30.4 ± 8.4, 22-38 Controls: 63 (29 M) 31.3 ± 7.7, 23-39	DSM-IV for primary insomnia	PSQI: 8.6 ± 2.3	ND	Monitoring time: during daytime, ECG sampling rate: ND, Period of analysis: ND, Spectral analysis technique: ND
Cellini et al. [63]	Insomniacs: 13 (8 F) 24.4 ± 1.6, 20-28 Controls: 14 (6 F) 23.3 ± 2.5, 20-28	Research Diagnostic Criteria for primary insomnia, subjectively reported symptoms ± 6 months and (PSQI ≥ 5, ISI ≥ 11)	PSQI: 10.0 ± 2.0, AIS: 15.77 ± 3.27, SOL: 16.19 ± 16.42, WASO: 45.88 ± 30.17	87(±8)%—95(±3)%	Monitoring time: during daytime, ECG sampling rate: 512 Hz, Period of analysis: 3 min, Spectral analysis technique: FFT
Baglioni et al. [85]	Insomniacs: 21 (19 F) 22.8 ± 3.31, 18-30 Controls: 18 (12 F) 22.0 ± 2.64, 18-30	DSM-IV	ISI: 12.09 ± 3.32	ND	Monitoring time: during daytime, ECG sampling rate: ND, Period of analysis: ND clearly, Spectral analysis technique: NA
Peter et al. [47]	Insomniacs: 21 (18 F) 48.2 ± 10.4, 18-75 Controls: 21 (18 F) 48.5 ± 11.1, 18-75	DSM-IV and PSQI ≥ 5	PSQI range: 10-17 and sleep characteristics available for revision	ND	Monitoring time: during daytime, ECG and BP sampling rate: 200, Period of analysis: 5-7 min, Spectral analysis technique: FFT
Nelson et al. [86]	Insomniacs: 34 (18 F) 21.1 ± 4.9, 18-45 Controls: 38 (26 F) 20.5 ± 2.2, 18-45	DSM-IV and PSQI ≥ 5	PSQI: 9.5 ± 3.0, BDI: 10.3 ± 6.0. Available characteristics for revision	ND	Monitoring time: during sleep, ECG sampling rate: ND, Period of analysis: ND, Spectral analysis technique: NA
de Zambotti et al. [12]	Insomniacs: 8 (4 F) 23.3 ± 2.4, 20-26 Controls: 8 (5 F) 23.3 ± 3.2, 19-28	DSM-IV for primary insomnia and PSQI: ≥ 6, AIS ≥ 6, ISI: ≥ 11	PSQI: 9.6 ± 1.3, AIS: 9.6 ± 3.2, ISI: 13.4± 3.3 Available characteristics for revision	90.0(±4)%—95.0(±2)%	Monitoring time: during sleep onset, ECG sampling rate: 500 Hz, Period of analysis: 2.5 min, Spectral analysis technique: FFT
Tsai et al. [87]	Insomniacs: 19 (13 F) 22.9 ± 1.6, 20-25 Controls: 14 (5 F) 22.4 ± 1.1, 20-25	Subjectively reported difficulty of falling asleep ≥ 20-30 min for ≥ 3 days per week over 6 months and daytime consequences and PSQI: > 5. Defined as psychophysiological insomnia based on the objective finding	PSQI: 11.3 ± 2.2, SOL: 25.9 ± 40.9. Available sleep characteristics for revision	90.1(±8.7)%—94.8(±2.8)%	Monitoring time: during sleep onset, ECG sampling rate: 500 Hz, Period of analysis: NA, Spectral analysis technique: NA
Maes et al. [71]	Insomniacs: 17 F 36.2 ± 9.6, 19-53 Controls: 11 F 37.6 ± 12.6, 21-59	DSM-IV-TR and subjective SOL > 30 min, at least 5 nights per week	SOL: 33.8 ± 17.1, Mean duration of insomnia 10.8 ± 10.9 years. Available sleep characteristics for revision	79.1(±10.1)%—89.0(±6.8)%	Monitoring time: during sleep onset and first NREM cycle, ECG sampling rate: 1000 Hz, Period of analysis: 5 min, Spectral analysis technique: FFT
Rothenberger et al. [72]	Insomniacs: 19 F 52.1 ± 2.2, ND Controls: 146 F 52.1 ± 2.2, ND	Self-report Insomnia Symptom Questionnaire (Incorporated to define DSM-IV criteria for insomnia and Research Diagnostic Criteria)	ND	ND	Monitoring time: during sleep, ECG sampling rate: 1024 Hz, Period of analysis: 2 min, Spectral analysis technique: FFT
Schramm et al. [57]	Insomniacs: 50 (27 M) 46.4 ± 8.6, 30-63 Controls: 36 (17 M) 44.5 ± 8.7, 30-63	DSM-IV for primary insomnia	PSQI: 12.11 ± 2.69	83.2(±10.1)%—89.1(±6.7)% (from the 2nd night)	Monitoring time: during sleep, ECG sampling rate: ≥ 200 Hz, Period of analysis: 8.5 min, Spectral analysis technique: FFT
Israel et al. [73]	Insomniacs: 54 (30 F) 34.6 ± 9.7, ND Controls: 22 (19 M) 26.5 ± 7.3, ND	DSM-IV for primary insomnia	SOL: 26.1 ± 18.3, WASO: 41.4 ± 33.5	85.6(±8.0)%—90.5(±5.5)%	Monitoring time: during sleep, ECG sampling rate: 1024 Hz, Period of analysis: 2 min, Spectral analysis technique: Autoregressive Model

Abbreviations— AIS: Athens Insomnia Scale, BDI: Beck depression inventory, CBT: Cognitive behavioral therapy, DSM: Diagnostic and Statistical Manual of Mental Disorders, ECG: electrocardiogram, ESS: Epworth Sleepiness Scale, F: female, FFT: fast Fourier transform, ICSD: International Classification of Sleep Disorders, INSD: Insomniacs with normal sleep duration, ISI: Insomnia Severity Index, ISSD: Insomniacs with short sleep duration, IV: 4th edition, M: male, min: minutes, NA: not applicable, ND: not defined, NREM: Non-rapid eye movement sleep, PSG: polysomnography, PSQI: Pittsburgh Sleep Quality Index, R: revised, SE: sleep efficiency, sec: second, SOL: Sleep onset latency, TR: text revised, TST: Total sleep time, WASO: Wake after sleep onset.

**Table 5:** Characteristics of the eight interventional studies.

Authors	Sample, Sex Age (mean±std, range)	Diagnostic criteria	Follow-up period Study type	Intervention	Insomnia Severity Sleep Efficiency	Monitoring and analysis details
Jobert et al. [88]	16 (4 M) (66.7 ± 5.8, ND)	ICSD for chronic or subcronic psychophysiological insomnia	ND placebo-controlled, randomized, 3-fold crossover	bezodiazepin lormetazepam (1mg) cyclopyrrolone zopiclone (7.5mg) placebo	ND	Monitoring time: during sleep, ECG sampling rate: 200 Hz, Period of analysis: averaged over 30 sec segments, Spectral analysis technique: NA
Lo et al. [74]	18 (7 M) (43.2 ± 15.4, ND)	Subjective complaints of difficulty initiating sleep and/or maintaining sleep for ≥ 3 months	4 weeks after the completion of dose titration open-label	Gabapentin Mean dose 540 mg Range 200-900 mg	Before: PSQI: 13.54^⋄^ 80.00% After: PSQI: 7.67^⋄^ 87.17%	Monitoring time: during sleep, ECG sampling rate: 400 Hz, Period of analysis: 10 min, Spectral analysis technique: ND
Chung et al. [64]	Responders: 16 (6 M) (57.9 ± 10.9, ND) Non-responders: 10 (4 M) (59.4 ± 7.4, ND)	ICSD-2	8-week period after the beginning of CBT open-label	CBT of 4 sessions, one every other week over an eight-week period	Responders- Before: ISI: 19.5 ± 4.0 77.7 ±18.9 After: ISI: < 8 ND Non-responders- Before: ISI:18.1 ± 4.9 70.8 ± 26.5 After: ISI: > 8 ND	Monitoring time: during daytime, ECG sampling rate: ND, Period of analysis: 5 min, Spectral analysis technique: ND
Jarrin et al. [75]	65 (22 M) (51.8 ± 10.0, ND)	DSM-IV-TR and ICSD-2 for chronic insomnia	6-week period after the beginning of CBT open-label	CBT, frequency of sessions was not specified	Before: ISI: 17.0±4.0 Subjective: 69.4±15.5% Objective: 80.1±12.1% After: 8.8±3.5 Subjective: 83.8±10 Objective: 88.6±8.4	Monitoring time: during sleep, ECG sampling rate: ND, Period of analysis: 2 min, Spectral analysis technique: FFT
Litscher et al. [76]	28 (5 M) (41.9 ± 14.6, 22-82)	Self-presentation at the hospital due to insomnia. AIS ranged from 6-21	10 min before, 20 min during, and 10 min after stimulation of the Shenmen acupoint open-label	Acupuncture by using the Shenmen acupuncture point on the left wrist	Before: AIS: 12.4±3.6 ND After: ND ND	Monitoring time: during daytime, ECG sampling rate: 4096 Hz, Period of analysis: 5 min, Spectral analysis technique: ND
Wang et al. [77]	31 (6 M) (54.3 ± 10.6, 39-82)	Self-presentation at the hospital due to insomnia and AIS ≥ 7 and a range of 7-22	10 min before, 20 min during, and 10 min after stimulation of the Shenmen acupoint open-label	Acupuncture by using the Shenmen acupuncture point on the left ear	Before: AIS: 14.7±4.4 ND After: ND ND	Monitoring time: during daytime, ECG sampling rate: 4096 Hz, Period of analysis: 5 min, Spectral analysis technique: ND
Chien et al. [67]	Experimental group: 34 F (51.09 ± 3.73, 45-55) Control group: 33 F (50.85 ± 3.73, 45-55)	Primary insomnia (definition not specified and Chinese version of PSQI (CPSQI) > 5	at the 4th week during treatment, and after 12 weeks of treatment cohort study	lavender aromatherapy	Experimental group- Before: CPSQI: 9 (8,12)^†^ ND After: CPSQI: ND ND Control group- Before: CPSQI: 11 (9,13)^†^ ND After: CPSQI: ND ND	Monitoring time: during daytime, ECG sampling rate: ND, Period of analysis: 3 min, Spectral analysis technique: ND
Tsai et al. [78]	Patient group: 14 ND (22.50 ± 1.22, 20-25) Control group: 14 ND (23.07 ± 1.64, 20-25)	Self-reported insomniacs who met the DSM-IV criteria for primary insomnia and PSQI > 6	2 days later after intervention (with a 1 week difference of each other) open-label	Controlled respiration at a slow frequency rate of 0.1 Hz, and a forced rate of 0.2 Hz during daytime rest	Patient group: Before: PSQI: 11.21±1.97 89.55±9.89 After: PSQI: ND 94.38±3.76* & 87.92±12.77** Control group: Before: PSQI: 2.93±1.33 94.99±2.88 After: PSQI: ND 94.47±2.80* & 93.48±3.24**	Monitoring time: during daytime and sleep, ECG sampling rate: 500 Hz, Period of analysis: 64 secs, Spectral analysis technique: FFT

Abbreviations— AIS: Athens Insomnia Scale, CBT: Cognitive behavioral therapy, CPSQI: Chinese Pittsburgh Sleep Quality Index, DSM: Diagnostic and Statistical Manual of Mental Disorders, F: female, FFT: fast Fourier transform, ICSD: International Classification of Sleep Disorders, ISI: Insomnia Severity Index, M: male, min: minutes, NA: not applicable, ND: Not defined, PSQI: Pittsburgh Sleep Quality Index, sec: second

*: Paced breathing at 0.1 Hz

**: Paced breathing at 0.2 Hz

^⋄^: global score

^†^: Median (Interquartile Range) instead of mean value and standard deviation

### Observational studies

#### Studies without specific diagnostic criteria for insomnia disorder

In studies without specific diagnostic criteria for insomnia disorder, physiological differences during sleep were investigated between the “poor” and “good” sleeper groups [79], as well as subjects with sleep-onset insomnia [80, 81]. Monroe [79] reported significantly higher HR 30 minutes before sleep in “poor” sleepers as compared to “good” sleepers. During sleep, HR of the “poor” sleepers was slightly, but not significantly, higher. Freedman et al. [81] found that sleep-onset insomniacs had increased HR prior to sleep, but not during sleep. Similarly, Haynes et al. [80] reported that sleep-onset insomniacs showed a mean HR 4.6 beats per minute, significantly higher than that of non-insomniacs, whilst the effects of pre-sleep cognitive stress were examined. Haynes et al. [80], concluded that insomniacs show higher levels of physiological arousal compared to non-insomniac subjects.

#### Nocturnal cardiovascular activity

In 1994, Stepanski et al. [82] assessed physiological activity in patients with objectively documented insomnia using specific American Sleep Disorders Association diagnostic criteria [90]. Subjects with chronic insomnia and normal sleepers slept in the laboratory overnight and were given a stressful performance task in the morning. HR was assessed for all participants before sleep, during sleep, and in response to acute stress. Nocturnal HR was significantly higher in insomniacs. The morning after, no difference was found in HR between the two groups, but HR was significantly higher during the stressful performance task in insomniacs. These results, regarding increased nocturnal HR, were confirmed by Bonnet et al. [11]. In particular, sleep and ECG measures were evaluated in insomnia patients and matched controls. In this study, spectral analysis of HRV revealed significant increases in LF and LF/HF and a decrease in HF in insomniacs compared to controls. Those changes were present across all sleep stages.

Recently, de Zambotti et al. [59] investigated nocturnal cardiovascular modifications in primary insomniacs compared to healthy controls, focusing on cardiac autonomic functioning. They found a significant, constant shorter PEP during all sleep stages of the insomniac group compared to good sleepers. In addition, they found the presence of short PEP to be directly associated with low quality of sleep assessed by the Pittsburgh Sleep Quality Index (PSQI) and by the Athens insomnia scale (AIS). In another study by de Zambotti et al. [60], the authors further studied ANS functioning in insomniacs and confirmed their previous results [59] regarding shorter PEP during all sleep stages in insomniacs. However, no significant differences in vagal activity (expressed by: the standard deviation of RR or NN intervals for a desired period (SDNN); the square root of the mean squared differences of successive NN intervals for a desired period (RMSSD); and the percentage of successive NN intervals that differ more than 50 ms (pNN50)) were reported between insomniacs and controls. Moreover, pre-sleep RR intervals duration (over a 5-min window) was positively associated with sleep efficiency (SE) and negatively associated with wake after sleep onset (WASO) in insomniacs. Spiegelhalder et al. [13] aimed at investigating the association between insomnia and alterations in polysomnographically determined nocturnal HR and HRV. In the insomnia group, results showed a lower wake-to-sleep HR difference compared to controls. The SDNN, was also significantly lower in the insomnia group. The authors [13] characterized the initial group of patients as subjectively reported insomniacs and they split up the insomnia group according to SE values. Thus, when restricting their analysis to insomnia patients with objectively determined short sleep duration (SE<85%), they found decreased HF, as well as decreased RMSSD and pNN50 values. Furthermore, Yang et al. [68] studied the long-term diurnal profile of HRV between insomniacs and healthy controls. In this study, HRV measures between awake and bed time were compared. The analysis of complexity indexes of HR dynamics using multiscale entropy derived measures, showed a considerable decrease in complexity during nighttime in insomniacs, compared to healthy subjects [68].

Schramm et al. [57] measured CPC in subjects with insomnia compared to good sleepers. In insomniacs, they found a lower HFC and a higher LFC on both nights on which subjects were polysomnographically monitored. In addition, HFC/LFC ratio was lower in both nights for insomniacs compared to controls reflecting poorer sleep quality. According to the authors HFC represents a marker of stable sleep while LFC represents a marker of unstable sleep.

Some studies [8, 53, 69, 70], however, have failed to provide differences between insomniacs and controls over all these cardiovascular measures. In these studies, the cardiovascular differences are either not seen during all sleep stages or not confirmed for all studied parameters. For instance, Bianchi et al. [53] when comparing long-term correlations and complexity of the HRV (using sample and multiscale entropy, Lempel-Ziv complexity, detrended fluctuation analysis, 1/f slope) during the night did not find significant differences except for a decreased sample entropy (m = 2, r = 0.2). In another study by Jurysta et al. [69], HF, LF, and LF/HF analyzed from the first three non-rapid eye movement (NREM)-rapid eye movement (REM) cycles did not reveal any significant differences between chronic insomniacs and healthy controls. Similarly, no differences in nocturnal LF, HF, LF/HF power were found by Farina et al. [8] during all sleep stages. In this study, 24 hours of ambulatory (home-based) polysomnography (PSG) were recorded. Patients showed modifications of HR (increased) and (increased normalized low frequency power (LF_*norm*_)), consistent with increased sympathetic activity, while awake before sleep and during early-stage-N2 (occurring in the first part of the night). No significant differences between insomniacs and controls could be found during slow-wave sleep (SWS), REM sleep, and, post-sleep wake. This study raises important questions regarding the interpretation of the ANS meaning of HRV features. For example, the authors found an increased RMSSD, LF_*norm*_, and LF/HF in the early-stage N2 in the insomniac group. Using the traditional interpretations that an increase in HR and the LF/HF ratio are linked with increased sympathetic activity, seem to be in contradiction with the increase in RMSSD which is often linked to an increase in parasympathetic activity [8]. In another study by Mazza et al. [70] sleep modifications induced by chronic benzodiazepine (BDZ) abuse in chronic insomniacs were evaluated. In this study, six insomnia patients affected by chronic BDZ abuse were compared to fifty five normal controls. No significant differences were found in LF, LF/HF power between BDZ abusers and controls, with the exception of an increased HF component. In addition, authors reported that abusers, compared to controls, had significantly lower indexes of electroencephalographic (EEG) arousal in all sleep stages and lower indexes of NREM sleep instability.

In the area of BP measurements during sleep in insomniacs, the literature is very limited. Lanfranchi et al. [83] investigated the 24-hour profile of arterial BP (brachial cuff arterial) in subjects with chronic primary insomnia, and tested the hypothesis that these subjects have higher nighttime BP, and an attenuation of nocturnal BP dipping compared to good sleepers. The authors documented significantly higher systolic BP and decreased systolic pressure dipping across the night in primary insomnia patients, compared to controls. However, no significant differences were observed in the HR and PSG-based profiles (with the exception of fewer periodic limb movements observed in the insomnia group) between insomniacs and good sleepers.

#### Cardiovascular activity during daytime

Several studies have been focused on the diurnal cardiovascular activity in insomnia population [47, 61, 62, 65, 66, 84]. Fang et al. examined the differences in HRV and daytime functioning. Five-minute recordings of ECG under paced breathing were obtained from all participants who were resting in a supine position. Authors found an increasing trend in LF/HF ratio in insomniacs compared to controls, but the differences between the two groups did not reach statistical significance [65]. Varkevisser et al. [61] measured cardiovascular parameters (average HR, RMSSD, PEP) in a group of subjects with chronic insomnia under strictly controlled constant-routine conditions with continuous wakefulness. Although physiologic indexes of arousal were slightly elevated in the insomnia group relative to the controls, the differences between the groups were not statistically significant. Floam et al. [84] investigated whether BP differs between individuals with insomnia disorder and healthy sleepers. Standard oscillometric BP measurements were collected in a seated position five times over a 15-min period during the day. Floam et al. [84] did not find any differences in BP measurements between individuals with insomnia disorder and healthy sleepers during daytime.

On the other hand, when Yang et al. [68] investigated the long-term diurnal profile of HRV, differences between insomniacs and healthy controls were seen. In this study, HRV measures between awake state and bed-time were compared. Compared to controls, insomniacs exhibited significant reductions in SDNN, HF, RMSSD, pNN50 during awake period. Alterations in LF/HF (increase), RMSSD (decrease), HF (decrease) were correlated with perceived sleep questionnaire score, suggesting that according to the authors, altered cardiac autonomic control and physiologic complexity is associated with poor sleep in patients with insomnia.

#### Cardiovascular activity during daytime while performing tasks

Several studies examined HRV during daytime while subjects performed a task [62, 63, 66, 85]. Covassin et al. [62] focused on cardiovascular re-activity to the task in primary insomnia. Cardiovascular re-activity is defined as the responsiveness of the cardiovascular system to react to a stressful task. The task was administered in two sessions, before and after a night of polysomnographic recording. Results of cardiovascular parameters showed higher HR and lower left ventricular ejection time values in insomniacs, as compared to controls in the evening. PEP was continuously reduced in insomniacs. Jiang et al. [66] aimed to examine HRV response to a postural change manoeuvre, in primary insomniacs and controls. HRV features were computed at the following times: seated rest and 0-5 min, 5-10 min and 10-15 min in the standing position. Jiang et al. [66] reported an attenuated or absent HRV response to postural change in primary insomnia subjects. Specifically, the increase in LF/HF ratio and the decrease in HF, reflected that parasympathetic predominance at rest shifted to sympathetic control while standing. However, this shift was much slower than in the normal controls, according to the authors. Furthermore, researchers [66] reported a significantly lower LF, SDNN, RMSSD, and HF in the primary insomnia group than in the normal control group. Two memory tasks and their association with cardiovascular activity were examined by Cellini et al. [63]. Compared to healthy controls, insomniacs exhibited shorter PEP, reduced HF and increased RPP at rest. Similarly, in another study [85] where psychophysiological reactivity to emotional stimuli using pictures (both related and unrelated to sleep) was examined, cardiac changes were observed in insomniacs compared to controls. According to the authors [85], enhanced cardiac vagal tone (defined as pulse-synchronized phase shifts in consecutive cardiac cycles) in response to all pictures, by people with insomnia compared to good sleepers was found, even though no significant effects were evidenced for HR responses between the two groups [85]. In another study by Nelson et al. [86], where the pre-sleep differential content of imagery and verbal thought was investigated, significant differences in HR were found between insomniacs and controls.

One study failed to report significant cardiovascular changes between insomniacs and healthy controls during tasks. Peter et al. [47] examined BRS using an exploratory protocol with paced breathing. The authors found no significant differences in BRS between insomnia patients and controls.

#### Sleep onset

Some studies investigated the autonomic changes that characterize wake-to-sleep transition. De Zambotti et al. [12] examined the cardiovascular activity during the switch from wakefulness to sleep in insomniacs. The cardiac activity was studied in baseline, as well as pre- and in post-sleep onset. Results showed higher initial HR (an index primarily modulated by parasympathetic activity at rest) in baseline in the insomniac group, but no differences between groups in pre- and post- sleep onset. In fact, HR showed a decrease in both groups during the transition from pre- to post-sleep onset. No significant differences were found between the two groups regarding HF_*norm*_. The most important result of this study, according to the authors, is the non significant changes in PEP values across sleep onset in the insomniac group. This result was interpreted as continuous, unchanged, sympathetic hyperactivation of insomniacs during sleep onset. In contrast, good sleepers showed the expected trend of increased PEP values during the transition from wakefulness to sleep. PEP was also significantly lower in insomniacs than in normal sleepers in both conditions (pre and post-sleep onset). These results agree with recent results from Farina et al. [8]. In this study, patients showed increased HR and LF_*norm*_, while awake before sleep and during early-stage-N2. In addition, Spiegelhalder et al. [13] reported that insomniacs have a lower wake-to-sleep HR reduction compared to controls. Sleep onset was also investigated on self-reported insomniacs. Tsai et al. [87] examined HR dynamics during the sleep onset period, between young self-reported insomniacs with long sleep latency, and normal controls. Linear regression and non-linear Hilbert-Huang transform of the HR slope were performed in order to analyze HR dynamics. Results indicated that a slower drop in HR dynamics during the sleep onset period seems to be a feature of sleep initiation difficulty. In addition, authors suggest that the magnitude of the change in HR during the sleep onset period is associated with the lengths of objective and subjective sleep-onset latency. In contrast to these studies, Maes et al. [71] failed to report any significant differences between insomnia female patients and controls preceding sleep onset, in either HR or HRV (LF, HF, and LF/HF) variables.

#### Cardiac activity and EEG

Some studies have evaluated cardiac autonomic tone in relation to sleep. For instance, Jurysta et al. [69] tried to determine the relation if chronic insomnia alters the relation between HRV and delta sleep EEG power. Results showed that the coherence between HF_*norm*_ and delta EEG was decreased significantly in insomniacs compared to healthy men. The authors suggested that the decreased coherence between relative vagal cardiac activity and delta sleep observed in patients with insomnia, in comparison to normal controls, could suggest a loss of control between brainstem structures, implied in cardiovascular and sleep controls [69]. Rothenberger et al. [72] examined whether EEG-HRV relationships in midlife women differ as a function of insomnia. They reported that time-varying correlations between delta EEG power and HF were stronger in women with self-reported insomnia, compared to healthy controls. Another study by Maes et al. [71] found an association between K-alpha (K-complex within one, second followed by 8–12 Hz EEG activity) in Stage2 sleep and a lower HF in SWS in female insomniacs. The authors interpreted the strong association found between K-alpha in Stage2 sleep and the lower HF as a state of hyperarousal continuing through sleep.

#### Stability of sleep and HRV

Israel and colleagues [73] focused on quantifying the short-term stability of multiple indices of sleep duration, continuity, architecture, and nocturnal physiology in good sleeper controls and insomniacs. Their results for HF and the LF/HF were similar for both insomniacs and healthy controls. Additionally, most quantitative EEG (QEEG) bandwidths, and HRV during sleep, show high short-term stability in good sleepers and patients with insomnia alike. According to the authors, one night of data is sufficient to extract reliable estimates of the examined outcomes in studies focused on group differences, or correlates of QEEG and/or HRV.

Table 6 presents a summary of the findings of the observational studies.

**Table 6:** Cardiovascular features in insomniacs compared to controls.

Authors	HR	BP	pNN50	SDNN	RMSSD	PEP	RPP	TP	VLF	LF	LF/HF	HF	LZC	CPC	SampEn	MSE	DFA	1/f	BRS-*α*	HF^†^-*EEG*_*δ*_	HF-EEG_(*K* − *α*)_
Stepanski et al. [82]^□^	↑																				
Bonnet et al. [11]^□^	↑			↓						↑^†^	↑	↓^†^									
de Zambotti et al. [60]^□^			NS	NS	NS	↓		NS			NS	NS^†^									
de Zambotti et al.[12]^△^	↑					↓						NS^†^									
Spigelhalder et al. [13]-^*subj*^^□^	NS		NS	↓	NS						NS	NS									
Spigelhalder et al. [13]-^*obj*^^□^	NS		↓	↓	↓						NS	↓									
Yang et al. [68]-^*bt*^^□^	↑		NS	NS	NS				NS	↓	NS	NS				↓					
Yang et al. [68]-^*dt*^^⋄^	NS		↓	↓	↓				↓	↓	NS	↓				NS					
Schramm et al.[57]^□^														↓^*HFC*^, ↓^*HFC*/*LFC*^, ↑^*LFC*^							
Bianchi et al.[53]^□^													NS		↓	NS	NS	NS			
Jurysta et al.[69]^□^								NS^b^*		NS^b^*	NS^b^*	NS^b^*								↓^*coh*^	
Farina et al. [8]^□^	↑^*w*,*N*2^		NS	↑^*esN*2^	↑^*esN*2^					↑^*w*,*esN*2^	↑^*esN*2^	NS									
Mazza et al. [70]^□^	NS									NS^†^	NS	↑^†^									
Lanfranchi et al. [83]^□^	NS	↑																			
Fang et al. [65]^⋄^									NS^*ln*^	NS^*ln*^	NS	NS^*ln*^									
Varkevisser et al. [61]^⋄^	NS				NS	NS															
Floam et al. [84]^⋄^		NS																			
de Zambotti et al. [59]^□^	NS					↓				NS^*b*^	NS^*b*^	NS^*b*^									
Jiang et al. [66]- seated^⋄^			NS	↓	↓				NS	↓	NS	↓									
Jiang et al. [66]- postural change^⋄^			NS	NS	NS				NS	↓	NS	↑									
Cellini et al.[63]-at rest^⋄^	NS^*log*^	NS^*log*^				↓^*log*^	↑^*log*^					↓^*log*^									
Cellini et al.[63]-at ESMT^⋄^	NS^*log*^	NS^*log*^				↓^*log*^	NS^*log*^					NS^*log*^									
Cellini et al.[63]-at NBT^⋄^	NS^*log*^	NS^*log*^				NS^*log*^	NS^*log*^					NS^*log*^									
Covassin et al. [62]^⋄^						↓															
Baglioni et al.[85]^⋄^	NS																				
Nelson et al.[86]^⋄^	↑																				
Peter et al.[47]^⋄^		NS^*LF*_*BP*_^, NS^*HF*_*BP*_^		NS	NS			NS		NS	NS	NS							NS^*αLF*^, NS^*αHF*^, NS^*TF* − *BRS*^, NS^*αTotal*^		
Tsai et al.[87]^△^	NS, ↓^*ls*,*nls*^																				
Maes et al.[71]^△^	NS									NS	NS	NS									↓^*corr*^
Rothenberger et al.[72]^□^												NS^†^								↑^*corr*^	
Israel et al.[73]^□^											NS	NS^†^									

Abbreviations—b: in both absolute and normalized power, BP: blood pressure, bt: during bedtime, coh: Coherence function, corr: correlation, CPC: cardiopulmonary coupling, DFA: detrended fluctuation analysis of RR time series, dt: during daytime, EEG_*δ*_: EEG delta power, ELMT: easy letter memory task, esN2: early stage of N2 collected from the first sleep cycle, HF: high frequency (*ms*^2^), HR: heart rate, LF: low frequency (*ms*^2^), LF/HF: ratio of LF and HF, ln: natural logarithmic transformation applied, log: logarithmic transformation applied, ls: linear slope of HR, LZC: Lempel-Ziv complexity of RR time series, MSE: multiscale entropy, NBT: N-back task, nls: non-linear slope of HR, NS: non-significant difference between insomniacs and controls, N2: N2 sleep stage, N3: N3 sleep stage, PEP: pre-ejection period, pNN50: percentage of successive NN intervals that differ more than 50ms, REM: Rapid Eye Movement sleep, RMSSD: square root of the mean squared differences of successive NN intervals, RPP: rate pressure product, SDNN: standard deviation of NN intervals, SampEn: sample entropy, VLF: very low frequency (*ms*^2^), w: wake

^†^: power is represented in normalized units

*: first three NREM-REM cycles

^⋄^: cardiovascular activity during daytime

^□^: nocturnal cardiovascular activity

^△^: cardiovascular activity during sleep onset

1/f: slope of power law regression line, K-*alpha*: K-complex within one second followed by 8-12 Hz EEG activity

### Interventional studies

#### Pharmacologic treatment

Jobert et al. [88] compared the effect of pharmacologic treatment (benzodiazepine hypnotic lormetazepam and cyclopyrrolone hypnotic zopiclone) on HR activity in elderly patients with a diagnosis of psychophysiological insomnia. Their results showed that the relation between sleep and HR remained constant under the influence of a single dose of lormetazepam or zopiclone. Although both compounds significantly altered the distribution of sleep stages, no relevant changes in ECG activity were observed when the proportion of the different sleep stages was taken into account. Lo et al. [74] evaluated the benefits of gabapentin in the treatment of primary insomnia in patients, and the results disagree with findings reported my Jobert et al. [88]. All insomnia patients in this study [74] received gabapentin treatment for at least 4 weeks. HRV analyses showed a significant increase in HF_*norm*_ in N3 sleep after treatment. In addition, they found a significant decrease in LF/HF ratio and in LF_*norm*_ in N3 sleep after treatment. The authors interpreted these results as a possible increase in parasympathetic activity, which could be explained by the significant increase in the duration of N3 sleep which was also observed in this study.

#### Cognitive-behavioral therapy

Psychological and behavioral therapies are the first-line treatment for innsomnia disorder [91]. Two studies investigated whether successful non-pharmacological treatment of insomnia would affect cardiac autonomic activity. The first study by Chung et al. [64] included 26 insomniacs who underwent four non-pharmacological treatment sessions over an 8-week period. Non-pharmacological treatment included sleep hygiene, abdominal breathing, stimulus control therapy, and instruction in progressive muscular relaxation, paradoxical intention and cognitive therapy and instructions on guided imagery [64]. The authors [64] observed significant HRV changes in the cognitive behavioural therapy (CBT) responders’ group (after treatment insomnia severity index (ISI) < 8) but no changes in the non-responder group. The responders group showed decreased LF and increased HF, pNN50, and SDNN values. A recent study by Jarrin et al. [75] included 65 patients treated for chronic insomnia. Patients received CBT over a six week period, and change scores from pre- to post-treatment derived from the ISI, sleep diary, and PSG were used as indices of sleep improvement. The study found that sleep improvements following CBT for insomnia are related with reduced HF and a trend for higher LF/HF ratio. Despite a trend between improved insomnia symptoms and increased parasympathetic activation, no differences in HF and LF/HF ratio during either S2 or REM were observed between treatment responders (vs. non-responders) or remitters (vs. non-remitters). Under the interpretation that reduced HF and higher LF/HF are associated with a lowered parasympathetic activation, these results do not seem fully compatible with the interpretation that the decrease in LF and increase in HF, found by Chung et al. [64], are associated with a decreased sympathetic activation and increase in parasympathetic tone.

#### Complementary and alternative medicines

Even though complementary and alternative medicine (CAM) is not the standard treatment for insomnia, studies have shown that adults use some form of CAM to treat insomnia or trouble with sleeping [92, 93]. Our search identified the following categories: acupressure-acupuncture treatment, lavender aromatherapy and paced breathing.

Two studies by Litscher et al. [76] and Wang et al. [77] tested the effect of arm acupuncture and ear acupressure on HR and HRV. Both studies reported that HR decreased significantly during and after acupuncture-acupressure stimulation. LF and HF band power increased significantly after the stimulation compared to baseline values [76], while LF/HF ratio did not show significant changes [76, 77]. Authors from both studies suggested that their results could serve as a basis for further investigations of auricular point stimulation for non-invasive complementary use in treating insomnia.

Chien et al. [67] evaluated the efficacy of lavender aromatherapy on HRV before and after twelve weeks of treatment, in midlife women with insomnia. ECG recording was performed at the baseline measurement, in the 4th and after the 12th weeks of follow-up period, in the experimental and control groups. Results showed a significant decrease in mean HR and increased SDNN and RMSSD after 20 minutes of lavender inhalation. Moreover, HF was also increased, while LF and LF/HF ratio remained unchanged. Lavender aromatherapy does not appear to have a lasting effect on HRV alterations in the long-term follow-up, even though the insomnia group experienced an improvement in the quality of their sleep.

Tsai et al. [78] aimed at investigating if breathing exercises are an effective behavioural intervention for insomnia. In this study, HRV under controlled respiration at a slow frequency rate of 0.1 Hz, and a forced rate of 0.2 Hz during daytime rest was assessed, for both young self-reported insomniacs and healthy controls. Authors reported reductions in HF during daytime period, and increased total power of HRV (between 0.0 and 0.4 Hz) in insomniacs compared to controls. In addition, changes in the sleep pattern and subjectively reported sleep quality observed after paced breathing at 0.1 Hz were observed. SE was increased if insomniacs practiced slow, paced breathing exercises for 20 minutes before going to sleep.

Table 7 presents a summary of the findings of the interventional studies.

**Table 7:** Cardiovascular features in insomniacs after intervention.

Authors	HR	pNN50	SDNN	RMSSD	Total Power	VLF	LF	LF/HF	HF
Jobert et al. [88]^□^	NS								
Lo et al. [74]^□^					↑^*N*2^	↑^*N*2^	↓^*b*,*N*3^	↓^*N*3^	↑^*b*,*N*3^
Chung et al. [64]^⋄^-responders	NS	↑	↑	NS	↑	↑	↓	NS	NS
Chung et al. [64]^⋄^-non-responders	NS	NS	NS	NS	NS	NS	NS	NS	NS
Jarrin et al. [75]^□^								NS	↓^*S*2,*REM*^
Litscher et al. [76]^⋄^	↓				NS		↑	NS	↑
Wang et al. [77]^⋄^	↓				NS			NS	
Chien et al. [67]^⋄^	↓		↑	↑	NS	NS	NS^*b*^	NS	↑, NS^†^
Tsai et al. [78]^□^^⋄^					↑^0.2 *Hz*^				

Abbreviations—b: in both absolute and normalized power, HF: high frequency (*ms*^2^), HR: heart rate, LF: low frequency (*ms*^2^), LF/HF: ratio of LF and HF, NS: non significant difference compared to before intervention, N2: N2 sleep stage, N3: N3 sleep stage, pNN50: percentage of successive NN intervals that differ more than 50ms, REM: Rapid Eye Movement sleep, RMSSD: square root of the mean squared differences of successive NN intervals, SDNN: standard deviation of NN intervals, VLF: very low frequency (*ms*^2^), w: wake

^†^: power is represented in normalized units

^⋄^: cardiovascular activity during daytime

^□^: nocturnal cardiovascular activity

0.2 Hz: paced breathing exercises at 0.2 Hz breath rates

## Discussion

### Summary of main results

#### Observational studies

The results of the observational studies included in this review offer little evidence of a single and consistently reported cardiovascular pattern among patients with insomnia when compared to healthy controls. However, some consistent findings can be drawn from the results. Results from several studies suggest alterations in cardiovascular autonomic control [11–13, 57, 59, 60, 62, 63, 66, 68, 86] and physiologic complexity [53, 68] in insomniacs. An increase in LF and/or LF/HF, sometimes in combination with a decrease in HF and/or SDNN, RMSSD, pNN50 (interpreted as parasympathetic activity) and shorter PEP, was interpreted as an increased sympathetic activity. This increased sympathetic activity was observed during sleep [11, 59, 60], daytime [62, 63], and during sleep onset [8, 12] in insomniacs compared to controls. Furthermore, changes in cardiovascular autonomic control were made evident through decreased parasympathetic activity during sleep [11, 13] and daytime [63, 66, 68]. Nonetheless, altered sympathovagal balance in insomniacs, compared to controls, was only reported by two studies. One study reported altered sympathovagal activity during sleep [11], and one during daytime while performing tasks [63], whilst four studies found no significant differences between insomniacs and controls in any cardiac measure [47, 61, 65, 73].

All of the studies that investigated the association of cardiac activity and EEG power [69, 71, 72] reported an altered relation between autonomic activity and EEG parameters in insomniac patients.

Two studies examined BP differences between insomniacs and controls. Lanfranchi et al. [83], while recording beat-to-beat non-invasive BP, reported an increased nighttime BP and an attenuation of nocturnal BP dipping in insomniacs. Floam et al. [84] found no differences between insomniacs and controls during the day. The number of studies examining BP in insomniacs compared to control is limited, thus making it unclear whether this behavior is characteristic of the disorder. In the literature, attenuation of nocturnal BP dipping in insomniacs was reported in a recent study by Sieminski et al. [94], where insomnics were compared to narcoleptic patients. Sieminski et al. reported that “non-dipping” is equally frequent in narcoleptic patients and patients with insomnia. It was suggested that observed abnormalities in circadian changes of BP values are caused by disturbed sleep architecture, rather than by deficiency of hypocretin [94].

#### Interventional studies

The studies included in this review reveal a wide range of intervention modalities and duration of intervention that was applied for insomnia treatment. Therefore, a comprehensive and unanimous conclusion regarding insomnia treatment, and cardiovascular alterations may not be expected. For instance, intervention practices consist of pharmacological, CBT, acupuncture, lavender aromatherapy, and paced breathing treatments. Even within each type of intervention, (e.g. pharmacological), different hypnotics have been used. As a result, different outcomes may be expected since different durations of intervention have been applied. This, in combination with divergent and heterogeneous insomniac groups, makes the evaluation of impact of interventions in cardiovascular characteristics in insomnia disorder challenging. Pharmacological studies [74, 88] do not present consistent results. The results from the two CBT studies [64, 75] included also do not suggest unanimous results. Studies that applied alternative intervention, such as acupuncture and aromatherapy, showed significant autonomic changes between insomniacs and controls, although no long-lasting or long-term follow up effect was found. Interestingly, several studies reported decreased LF [64][74], and LF/HF [74], which could be interpreted as sympathetic activity and increased HF [64, 74, 87], pNN50 [64], and SDNN [64], interpreted as enhanced parasympathetic activity after intervention. In these studies, responders to intervention present contradicting results compared with those reported by observational studies (especially those with PSG-defined insomniacs). Thus, these treatment outcomes may provide additional evidence of the hyper-arousal concept [7]. Nevertheless, we should note that in each intervention type, the number of existing studies is limited and therefore the impact of insomnia interventions on cardiovascular activity needs further thorough investigation within well-designed and controlled trials.

### Implications of the insomnia heterogeneity

Through careful examination of the reported results, and the testing protocols used in the examined studies, several explanations may be put forward to account for different expressions of cardiovascular alterations between insomniacs and controls. These explanations form different perspectives of the same hypothesis. The heterogeneity of the disorder, and the existence of different phenotypes, could explain the lack of a comprehensive and consistent result among studies.

As shown in Table 3, over the last decades there has not been a commonly used definition of insomnia. While all the studies to date have examined insomnia patients using quantitative criteria (often based on the diagnostic criteria defined in the DSM-IV or ICSD) the definitions used differ across studies. The populations tested in the above studies are disparate, ranging from a sample diagnosed with insomnia according to Disorder of Initiating and Maintaining Sleep (DIMS) (1979) [82], to a study using DSM-V criteria [4] combined with various severity requirements. The lack of strict and consistent diagnostic criteria increases the heterogeneity of the sample, and thus reduces the possibility of detecting consistent results across studies. In most instances, it appeared that findings and resulting opinions derived from a single interviewer using a standard or semi standard clinic interview [8, 78, 85, 86], served as the basis for diagnostic evaluation. In other studies, the number of interviewers was not clearly mentioned [12, 57, 59, 65, 66]. Evidence shows that standard clinic interviews, used in practice to diagnose insomniacs, have very modest inter-rater agreement [95], and Edinger et al. [2] highlighted the importance and need for a more reliable interview method for the research setting. Further complicating matters, the DSM-IV-TR gives four diagnostic sub-types for insomnia, whereas the ICSD-2 lists 11 sub-types. Considering that the diagnostic assessment of insomnia for the majority of the studies [8, 12, 53, 57, 59, 60, 62, 63, 65, 66, 68, 69, 71–73, 83–87] was relied on the clinical interviews, and the results of subjective questionnaires, it is not always feasible to differentiate between paradoxical and psychophysiological insomnia. A PSG recording during the night measures sleep duration and it can be used to exclude sleep misperception and other sleep disorders such as sleep-related movement disorders and apnea. However, only a limited number of studies [11, 13, 82] included PSG defined insomniacs since PSG is not recommended for the diagnosis. Portable solutions for monitoring sleep have a reduced number of channels and thus yield limited reliability for these issues, and was only used in two studies [47, 61].

All studies that used PSG-defined insomniacs with low SE (<85%) showed alterations in cardiac control, expressed by elevated HR [11, 13, 82], increased LF and LF/HF [11] (interpreted as sympathetic activity), and decreased HF [11, 13], pNN50 [13] (interpreted as parasympathetic activity). These studies restricted their selection to insomniacs with objectively short sleep duration, and a SE that is lower than 85%. Even though the number of studies including PSG-defined insomniacs is small, insomniacs with objectively short sleep duration appear to be the most biologically severe phenotype, as supported by other evidence presented in [96, 97]. Researchers state that insomnia with objective normal sleep duration is associated with cognitive-emotional, cortical arousal, and sleep misperception, but not with signs of activation of both limbs of the stress system, or medical complications. In another study by Vgontzas et al. [98], researchers used a cross-sectional design in order to investigate the relation between hypertension, insomnia, and polysomnographically determined sleep duration in 1741 participants. They found that only insomnia with short sleep duration was linked to hypertension. Interestingly, neither insomnia without short sleep duration, nor short sleep duration without any sleep complaints, were significantly associated with hypertension [98]. Another recent study by Miller et al. [99] demonstrated that both insomnia phenotypes based on objective sleep duration, can be identified without priori cut-offs of sleep duration, and through cluster analytic statistical techniques. This study provides further support for the insomnia with objective short sleep duration phenotype, and its associated physiologic mechanisms, such as sympathetic activation [97]. Additionally, autonomic dysfunction and the association between sympathetic nervous system activity and blood pressure have been identified as one of the indicators of cardiovascular risk [100]. Thus, evidence of hypertension [96–98] combined with the cardiovascular differences of ANS activity between insomniacs and controls reported in this work may offer a tool for detecting patients at greater cardiovascular risk.

It appears that several studies show that the patient group has a wide range of SE. In most studies, the standard deviation of sleep efficiency of the insomniac group is two (or more) times higher than the standard deviation of sleep efficiency of controls [11, 12, 57, 59, 60, 62, 63, 65, 69, 83, 87]. This result, in combination with the chronic aspect, development of the disorder over time and its severity, could be an expression of the heterogeneous insomnia group. Additionally, due to the fact that most of the examined studies were small, comparisons between the two groups depend on thorough subject selection, in order to maximize group differences, and minimize variability. However, in most studies, the normal control groups were not required to demonstrate a specific SE prior to admission to the study. In many studies, the average SE between insomniacs and controls did not differ substantially, and in many cases, SE of the control group was lower than 90% [13, 57, 61, 71]. Moreover, many studies did not provide information regarding the sleep efficiency of the participants from both groups [47, 53, 66, 68, 72, 84–86], whereas some studies only used actigraphy measures [61, 65]. Several studies indicate that actigraphy appears to underestimate wake, and thus overestimate sleep duration [101, 102]. Since insomniacs are shown to constitute such a heterogeneous group, even under specific selection criteria and laboratory conditions, the presence of different expressions of cardiovascular alterations between insomniacs and controls may occur.

Variability in medical and psychiatric screening of patients may also account for the different expressions of cardiovascular alterations found in the studies. For example, in some studies [65, 68, 84–87], the patient group did not undergo screening for comorbid sleep disorders, and medical and psychiatric illnesses, such as apnea, narcolepsy, major depression, etc. In addition, some studies included patients who: i) occasionally took central nervous system-active medication [61]; ii) had chronic benzodiazepine abuse [70]; or iii) stopped medication 1-2 weeks before the study [69]. It is known that medication such as benzodiazepine and antidepressants influence cardiovascular activity [103, 104]. Other studies included patients with insomnia and comorbidities, such as (mild) apnea [72][73], and major depression [86]. A recent prospective follow-up cohort study comprising adult patients with obstructive sleep apnea (OSA) from 17 European countries and Israel, showed that phenotypes with insomnia symptoms comprised more than half of OSA patients [105]. Given that many medical and psychiatric illnesses are known to have pronounced effects on sleepiness and nighttime sleep, the lack of adequate screening in either participant group may mask a true difference, or reflect different cardiovascular changes, between groups using these measures.

Additional factors contributing to the inconsistency of the expressions of cardiovascular alterations could be sex, age range, as well as the technique used for spectral analysis estimation and the period of analysis. Studies show that ANS function, and therefore HRV, declines with aging [106]. Other age-related changes, such as a decline in baroreceptor sensitivity, may result into compensatory ANS response, which could mask underlying functional deficiencies [106]. The very broad age range of participants in several studies [8, 47, 65, 69] considerably increases measurement variability in both groups, and decreases the possibility to show group differences. The HR variation for healthy subjects between 20 and 70 years, was studied by Bonnemeir et al. [107]. It was found that HRV decreases with age and HRV variation is more likely in the case of females than men. Some studies have investigated gender and age-related differences in time and frequency domain measures [108, 109] and non-linear components of HRV. Furthermore, a significant difference between day and night time, when studying HRV by means of spectral and time domain analysis, was highlighted in other studies [108, 110]. Regarding the spectral analysis of cardiovascular oscillations, studies have shown that frequency domain analysis based on parametric (AR) or nonparametric (FFT) methods do not always agree [111, 112]. In addition, changes in the cardiovascular markers observed during physiological conditions were not equally detected by AR and FFT [111]. The majority of the reviewed studies [12, 13, 47, 57, 59, 60, 63, 65, 69, 71, 72] used FFT for spectral analysis estimation while some studies [8, 70, 73] used AR. A further complicating factor is the large variation in the time segments chosen for the computation of the cardiovascular features, varying from a few seconds [61] to an hour [82]. Furthermore, a low accuracy in the estimation of the HRV features could have emerged due to physiological or technical artifacts during R-peak detection, since not all studies performed a visual check of RR intervals.

### Limitations

The review was carried out only on papers written in English. Therefore, possibly some relevant findings may have been missed. In addition, statistical meta-analysis of the results of the included studies was not feasible due to the lack of comparable population, analyses, and cardiovascular measures.

### Conclusion and suggestions

In conclusion, we found that in most of the observational studies (21 out of 26) differences in cardiovascular activity and HRV-EEG relation between insomniacs and controls were observed. Based on our review of the literature, we hypothesize that these differences in cardiovascular activity can be explained by the heterogeneity of the disorder. The various studies included in this review describe a heterogeneous insomnia group, including patients with comorbidities (e.g. depression, OSA), insomniacs who took medication, insomniacs suspected of misperception of sleep, and PSG-diagnosed insomniacs. Cardiovascular activity in the PSG subgroup with objectively measured short sleep duration (sleep efficiency <85%) tended to be more consistent than cardiovascular activity in non-PSG insomniacs. In the literature, studies show that this subgroup appears to be the most biologically severe phenotype [96, 97] and is linked to hypertension [98]. Therefore, we hypothesize that autonomic regulation tends to be consistent between insomniacs, as long as they are grouped into their respective phenotype, as shown the insomnia subgroup with objectively short sleep duration. When only including HRV as a measure for autonomic regulation, differences in autonomic activity between insomniacs and controls are not so evident among all studies, as also concluded by Dodds et al. [17], where nearly half of the total of 17 observational studies reported no significant HRV differences.

## Supporting information

S1 Appendix Search strategy details. (PDF)

S1 Table PRISMA checklist. (PDF)
