# Supplementary material for: Measures of cardiovascular autonomic activity in insomnia disorder: A systematic review
Source: PLoS One. 2017 Oct 23;12(10):e0186716. doi: 10.1371/journal.pone.0186716 (PMC5653329; doi:10.1371/journal.pone.0186716)
Supplement: S1 Appendix — (PDF) [file pone.0186716.s001.pdf]

Initially, PubMed database was used to identify relevant clinical studies designed to study cardiovascular differences between insomniacs and controls. There were no further restrictions regarding date of publication. For this purpose the following search was applied:

(Cardiac OR "heart rate" OR cardio OR autonomic OR sympathetic OR parasympathetic OR "blood pressure" OR arterial OR vascular OR baroreflex) AND insomni*

This search was performed in all fields and all articles were carefully and manually examined by the 1st author to ensure relevance regarding the inclusion criteria. Reference lists of relevant articles were also carefully reviewed. Results showed that relevant articles will be sufficiently identified by using the following search

(Cardiac OR "heart rate" OR cardio OR autonomic OR sympathetic OR parasympathetic OR "blood pressure" OR arterial OR vascular OR baroreflex) AND insomni*[Title]

Therefore, the search in Scopus was modified accordingly:

TITLE-ABS-KEY (cardiac* OR "heart rate" OR cardio* OR autonom* OR sympathet* OR parasympathet* OR "blood pressure" OR arteria* OR vascular* OR baroreflex) AND TITLE (insomni*) .
